# Anatomical Considerations to Optimize Sensory Recovery in Breast Neurotization with Allograft

**DOI:** 10.1097/GOX.0000000000001985

**Published:** 2018-11-07

**Authors:** Ivica Ducic, Joshua Yoon, Arash Momeni, Uros Ahcan

**Affiliations:** From the * Washington Nerve Institute, McLean, Va.; † Department of Surgery, The George Washington University, Washington, D.C.; ‡ Division of Plastic and Reconstructive Surgery, Stanford University Medical Center, Palo Alto, Calif.; § Plastic and Reconstructive Surgery, University of Ljubljana, Ljubljana, Slovenia, EU.

## Abstract

**Background:**

Breast numbness is a recognized problem following mastectomy and subsequent reconstruction. Contemporary literature acknowledges the positive role of breast neurotization, but it is characterized by a variety of technical approaches and substantial heterogeneity with respect to the degree of recovered sensibility that remains suboptimal in comparison with other sensory nerve reconstructions. This study’s purpose was to provide an anatomical basis for observed inconsistencies and therein provide a principle that can be used to develop a technical approach that will optimize sensory recovery.

**Methods:**

Anatomical dissections on 6 fresh cadavers, that is, 12 hemi-abdominal flaps and 12 hemi-chest dissections, were performed. The technical aspects of harvesting the abdominal flap with a nerve target, that is, inclusion of a sensory nerve branch only, recipient nerves in the chest, and the applications of allograft for acquired nerve gap reconstruction were investigated.

**Results:**

Abdominal flaps that include sensory-only intercostal nerve 10–12 segments and identification of recipient chest wall intercostal nerves 2–4 could be consistently performed. The dissection and extraction of the donor sensory nerve target allowed preservation of the motor rectus innervation. The acquired nerve gap was easily bridged by an interposing allograft, allowing free arch of rotation for flap inset, suitable for either single or dual neurotization.

**Conclusions:**

We provide a likely anatomical explanation for suboptimal sensory recovery after deep inferior epigastric perforator (DIEP) flap breast neurotization, as mixed intercostal autograft is prohibitive to maximal sensory recovery. Breast neurotization with allograft that bridges sensory donor intercostal nerves to sensory recipient intercostal nerves should anatomically optimize restoration of breast sensibility.

## INTRODUCTION

Postmastectomy breast reconstruction has evolved to become a fundamental element in breast cancer care.^1^ Although implant-based breast reconstruction is the most common reconstructive modality, autologous reconstruction has been shown to be associated with greater long-term patient satisfaction.^2^ However, despite significant technical advances in autologous reconstruction to minimize donor-site morbidity such as the development of perforator-based flaps, abdominal wall weakness and donor-site hernias remain significant complications.^2–9^

Interestingly, the abundance of reports that focus on donor-site morbidity is contrasted by the paucity of studies focusing on recipient-site outcomes beyond just flap survival and breast shape. The importance of breast sensation cannot be overstated as it has a tremendous impact on postoperative quality of life.^10^ In fact, the issue of postmastectomy loss of sensation has recently been prominently addressed in the mainstream media.^11^ Hence, patients are increasingly inquiring about modalities that not only reconstruct the breast mound but also restore sensation. A topic of much debate in this regard is breast neurotization by virtue of flap reinnervation/neurotization at the time of transfer.

Breast neurotization is not a novel topic and has been discussed in the literature since the early 1990s.^10,12–19^ Available evidence suggests that restoration of sensation is an important measure. Cases of involuntary thermal and mechanical injury have been reported with a resultant negative impact on patient-rated quality of life metrics.^12,17,20–25^ Yet, since the introduction of sensate flaps for breast reconstruction, the debate has centered on whether nerve coaptation is necessary for recovery of flap sensation or whether collateral ingrowth of nerve fibers is sufficient for meaningful sensation.^19^ However, multiple studies have shown that flap neurotization results in more expeditious and improved sensory recovery, improved patient satisfaction, and patient-reported quality of life.^10,17,24,25^ These observations are contrasted by few studies that have failed to demonstrate such a difference in outcomes.^17,26–28^

A recent comprehensive systematic review by Beugels et al.^29^ examined the sensory recovery of autologous flaps with and without nerve coaptation. Although the high degree of heterogeneity and lack of standardization between studies made comparative analysis impossible, the authors did state that “sensory recovery of innervated flaps is superior, starts earlier, and gradually improves over time with a higher chance of approaching normal sensation compared with noninnervated flaps.”^29^

However, although innervated deep inferior epigastric perforator (DIEP) flap reconstructions do result in a return of sensation, the amount of functional sensory recovery has been variable and poorer than what is expected. In the majority of the studies that evaluated sensory recovery after reinnervation, the sensory return of innervated flaps never reached normal values. The reconstructed breast was noted in some studies to be only half as sensitive as the contralateral native breast.^18,29^ To address this unexplained disconnect, the authors conducted this study to offer an anatomical explanation for the less than expected sensory recovery. In addition, evidence-based nerve gap reconstructive options were reviewed to suggest anatomically appropriate choice for bridging the acquired nerve gap, that would allow free arch of rotation for flap inset and remain suitable for either single or dual neurotization.

## MATERIALS AND METHODS

Anatomical dissections on 6 fresh female cadavers between the ages of 43 and 62 (average, 53 years), that is, 12 hemi-abdominal flaps and 12 hemi-chest dissections, were performed by a single investigator. Abdominal dissections included traditional DIEP flap exposures with purposeful identification and preservation of the intercostal nerves entering the flap at the anterior abdominal wall/rectus muscle interface, enabling us to trace the sensory nerve branches into the rectus abdominis. Chest dissections included the exposure and preservation of internal mammary arteries and intercostal nerves 2–4. The diameters of the exposed nerves were measured with a *REXBETI* electronic caliper that measures to the nearest 0.01 mm.

The technical aspects and mechanics of harvesting the abdominal flap with a nerve target, that is, inclusion of a sensory nerve branch only, recipient nerves in the chest, and specifics of abdominal intercostal donor and chest recipient nerves were investigated. In addition, technical aspects of allograft applications for acquired nerve gap reconstruction were also evaluated.

## RESULTS

### Cadaver Dissection

**Fig. 1.**
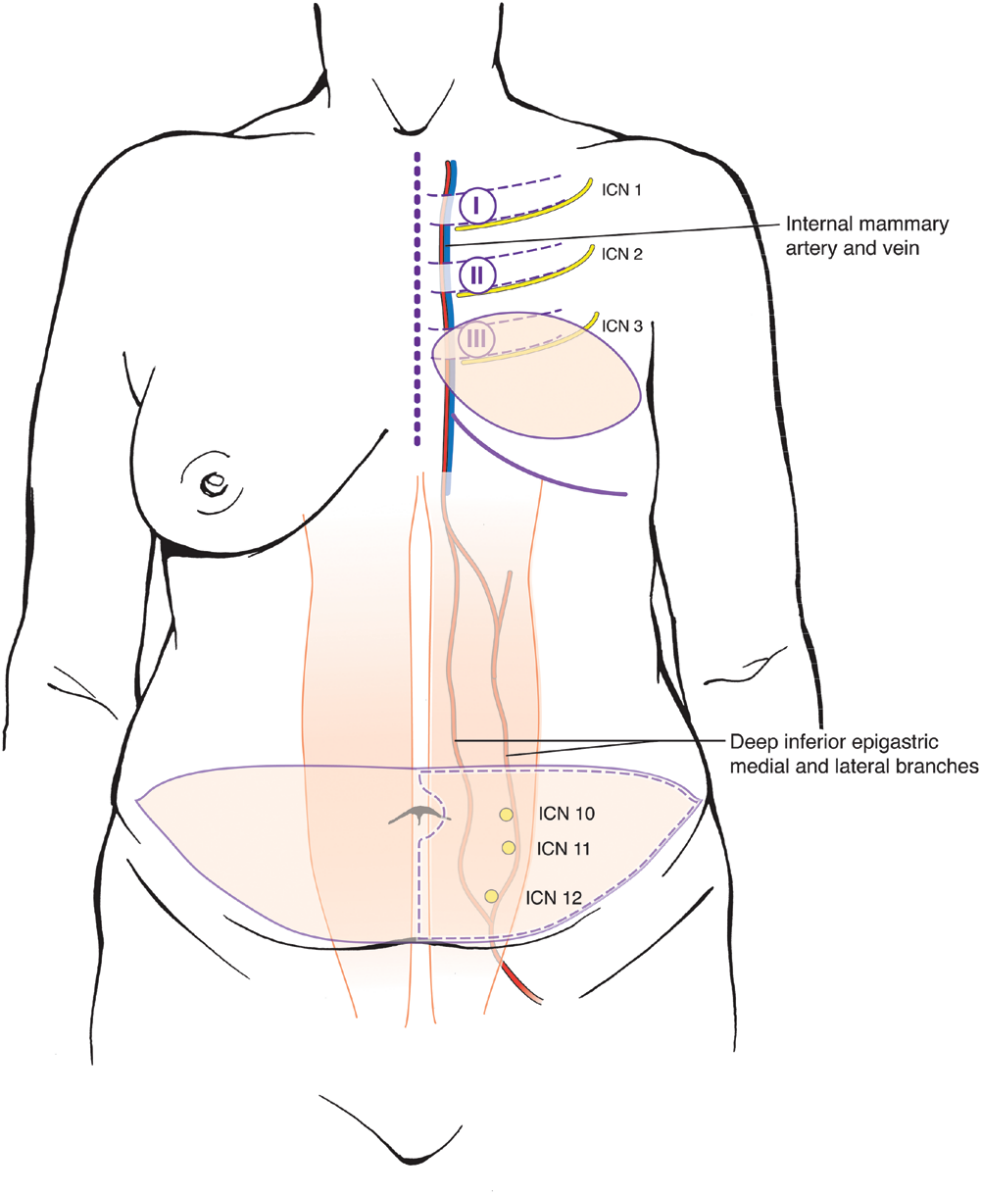
Key anatomical landmarks for DIEP flap breast neurotization with outlines of DIEP abdominal flap and postmastectomy chest wall defect. Essential nerves (ICN1, ICN2, ICN3, ICN10, ICN11, ICN12), vascular structures (medial and lateral deep inferior epigastric artery [DIEA] internal mammary artery, and vein), and bony landmarks (ribs I, II, III) shown.

**Fig. 2.**
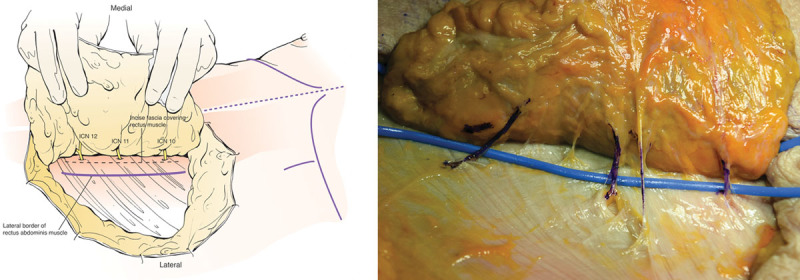
DIEP flap dissection in standard lateral to medial fashion. A, Schematic drawing demonstrating typical position of distal ends of the sensory components of respective intercostal nerves and expected incision of rectus sheath lateral to intercostal nerves. B, Blue-ink labeled lateral perforators and adjacent ICN10-12, and 1 additional sensory branch lateral to ICN11.

**Fig. 3.**
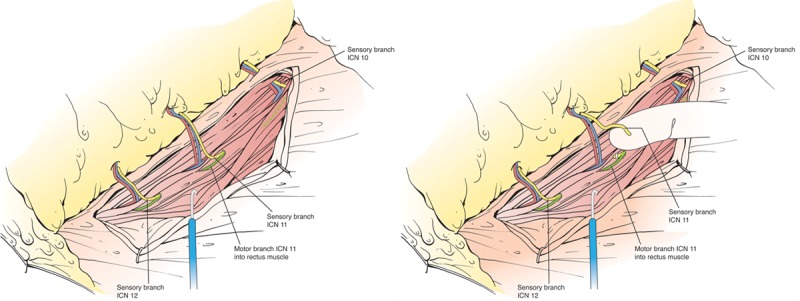
Exposure of the ICNs after the incision of anterior rectus sheath and spread of longitudinal rectus muscle fibers. A, Schematic representation of the retrograde dissection of sensory component of the intercostal nerves (yellow) until joining the motor components (green) at an intramuscular sensory-motor Y-junction. If medial row perforators were dominant and used for flap supply, lateral anterior rectus sheath fascial opening and rectus spread can be limited only to allow sensory ICN harvest. B, Separation of sensory component of ICN11 (yellow), just distal to Y-junction with preserved motor component (green) with longitudinally dissected rectus muscle.

Standard abdominal and chest anatomical landmarks for DIEP flap breast reconstruction are defined (Fig. 1). In all hemi-abdominal flaps, sensory nerve branches of the donor intercostal nerve 10–12 were identified piercing the anterior rectus sheath along the lateral perforators (Fig. 2). By opening the anterior rectus sheath, the branches could be traced in a retrograde fashion to a sensory-motor Y-junction, which is the division of the motor and sensory component of the respective intercostal nerve. By dividing the sensory branch of the intercostal nerve distal to the Y-junction, the motor component could reliably be preserved (Fig. 3).

Sensory branches of the donor intercostal nerves, although expected to follow the lateral perforators, did so only in 75% of cases, while the remainder pierced the anterior rectus sheath or external oblique fascia in various distances from the lateral perforators. Some of these branches were found even lateral to the very tip of the raised flap. The diameter of these sensory branches also widely ranged, averaging 1.23 ± 0.6 mm (0.5–2.4 mm) but with no particular pattern.

**Fig. 4.**
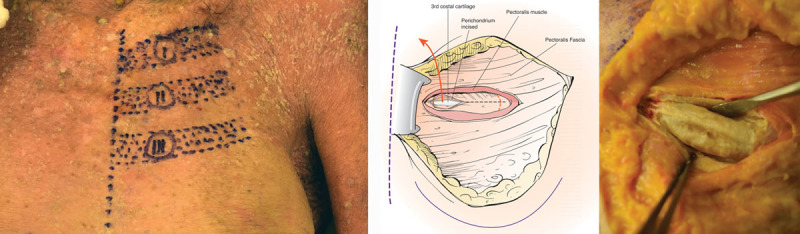
Dissection approach to third rib cartilage. A, Marking of projected 1–3 rib cartilage location. Dashed vertical line is sternum. B, Postmastectomy defect showing longitudinally spread pectoralis major muscle fibers and exposed third rib cartilage perichondrium. C, Perichondrium is incised and separated circumferentially, in preparation for the third rib cartilage for removal.

**Fig. 5.**
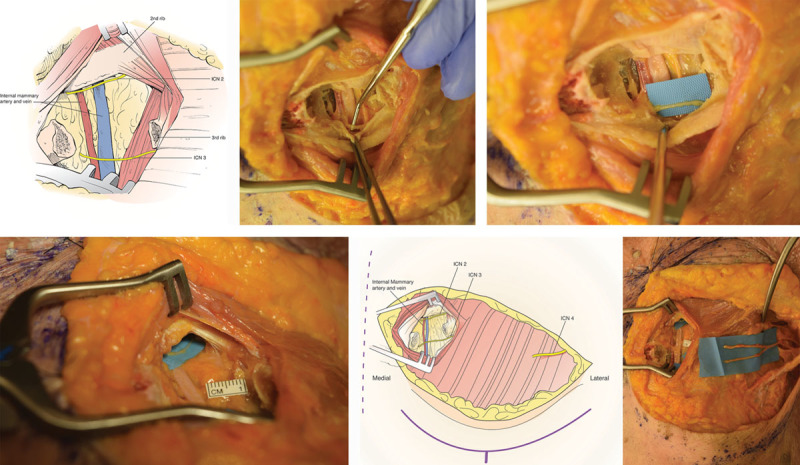
Identifying intercostal nerves. A, Schematic drawing showing internal mammary artery and vein, as well expected anatomical location of the ICN2 and ICN3. B, Anatomical dissection identifying ICN3 in its location along the inferior border of the third rib cartilage. C, More complete exposure of ICN3. D, Anatomical dissection of ICN2 after careful dissection from perichondrium and the inferior border of the second rib cartilage if dual innervation with ICN3 is desired. E, Schematic drawing showing internal mammary artery and vein, and dissected ICN2-4. F, Anatomical dissection identifying 2 branches of the laterally located ICN4, in addition to more medially located ICN2 & ICN3.

In all hemi-chest dissections, following mastectomy, the third rib cartilage marked, then accessed in standard fashion by spreading pectoralis major muscles along their natural path (Fig. 4). Upon third rib cartilage removal, the second and/or third intercostal nerves were dissected medially, at the site of cartilage removal (Fig. 5A–D). By tracing the nerves medially, intercostal nerves 2 and 3 were found to reliably course superficial to the internal mammary vessels. Although the second and third intercostal nerve displayed a constant course along the inferior border of the respective rib, the piercing point of the fourth intercostal nerve lateral to the pectoralis major muscle at the anterior axillary line randomly varied in our dissections (Fig. 5E, F). The average diameter of second and third intercostal nerve was 1.3 ± 0.4 mm (0.7–1.7 mm), while intercostal nerve 4 was larger 2.2 ± 0.4 mm (1.4–2.5 mm).

**Fig. 6.**
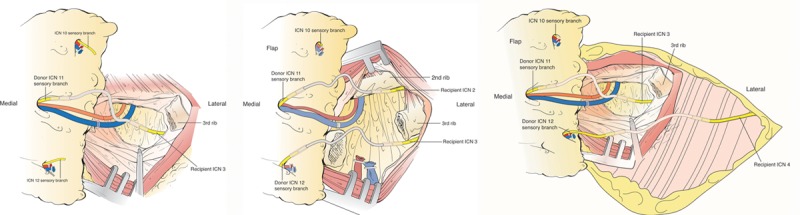
Bridging of the donor to recipient nerves with processed human nerve allograft. A, Schematic drawing showing tension free single nerve neurotization with ICN11 and ICN3 with coaptation of the nerve facilitated by translucent porcine intestinal submucosa nerve connector, as alternative to direct suture. B, Schematic drawing showing tension free dual nerve neurotization with ICN11 and ICN12 connected to ICN2 and ICN3, respectively. C, Schematic illustration of a tension free dual nerve neurotization with ICN11 to ICN3, and ICN12 connected to ICN4, respectively.

Following flap transfer to the chest, completion of the revascularization, and the dissection of recipient chest nerves, the acquired nerve gap defect varied in every dissection. The primary reason for the variability was because of the manipulation and insetting of the flap. Taking this into consideration, an interposing nerve allograft, either 5 or 7 cm in length allowed an unopposed flap rotation and flap inset in all dissections, while utilizing various ICN2-4 combinations (Fig. 6).

## DISCUSSION

Breast neurotization follows the general principles of standard nerve injury repair. When possible, tensionless primary repair should be performed; however, if primary repair is not possible, then bridging materials are utilized, which may include nerve autografts, tube conduits, and processed nerve allografts.^30–32^

**Fig. 7.**
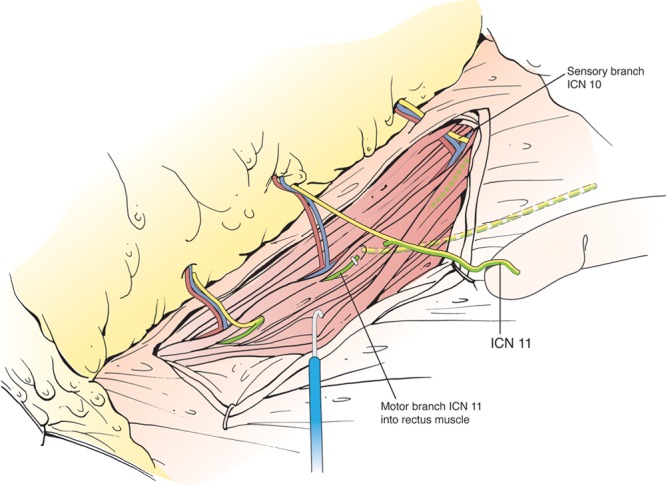
Traditional dissection and separation of donor intercostal nerve. Schematic drawing showing the extended donor pedicle that consists of both sensory (yellow) and motor (green) components that were dissected out and proximal to the rectus abdominis muscle (original position of pedicle illustrated by dashed yellow line).

Authors have commented on being able to raise a sufficiently long intercostal nerve (ICN) branch with the abdominal flap that allows for tensionless primary repair. Indeed, an intercostal nerve up to 10–12 cm in length can be harvested, but the harvested nerve would include both sensory and motor components (Fig. 7). However, in harvesting a mixed nerve lies the crux of the issue. Once the recipient intercostal nerve begins to regenerate toward the transferred flap by the donor mixed intercostal nerve, only the sensory half of the nerve may neurotize the flap, while the remaining half of the regenerating nerve would blindly end into the clipped donor motor component. We believe this is the anatomical basis and explanation as to why there is an unexpected shortcoming in the degree of sensory recovery in the autograft-neurotized breast. Additionally, donor-site morbidity (bulge and hernias due to iatrogenic rectus muscle denervation) is also increased in these instances, particularly if the large type 2 nerve as described by Rozen et al.^3,33–36^ is sacrificed.

Based on our anatomical dissections, we were able to consistently dissect and extract only the sensory branch of the ICN to the abdominal flap. By dividing the nerve branch distal to the sensory-motor Y-junction, motor innervation to the rectus abdominis muscle is preserved and the risk of axonal loss via divided motor side branches is minimized. Thus, donor-site morbidity is kept to a minimum and more importantly the chance for successful flap reinnervation is increased. This technique, however, results in a rather short target nerve, that in return mandates the use of techniques to bridge the resulting nerve gap.^9,37^

In terms of nerve gap bridging mediums, a mixed sensory-motor autograft for aforementioned reasons may adequately bridge the gap, but have less than adequate sensory recovery and the gap length during breast neurotization far exceeds what is recommended for reconstruction with nerve conduits, which is about 6 mm.^38–44^ The most comprehensive review on tube conduits and allografts as an alternative to nerve autografts was performed by Safa and Buncke^45^ in 2016 and they found that in gaps under 6 mm, tube conduits performed well, but beyond this length the reliability declined rapidly and outcomes were significantly less consistent. In light of these findings, the favorable breast neurotization results reported by Spiegel et al.,^18^ who used 40 mm hollow tube conduits are rather surprising and not otherwise replicated. In contrast, processed nerve allografts are found to perform reliably in gaps up to 70 mm with the difference being attributed to the structural preservation of the nerve architecture and the presence of laminin in the nerve micro-environment of the allograft.^46,47^ Collectively, the current clinical data show that allografts are safe, they result in successful neurotization for reconstructed nerve gaps up to 70 mm in length, their results are comparable with nerve autograft without the associated donor-site morbidity, and their clinical results are significantly better than hollow tube conduits.^39,40,46–49^ For example, Salomon et al.^47^ examined the use of allograft to span defects ≥ 50 mm of the inferior alveolar nerve and found that 87.5% of patients had sensory recovery to S3 or greater on the Medical Research Council Classification (MRCC) scale, which has been often used as the metric for functional sensory recovery.

**Fig. 8.**
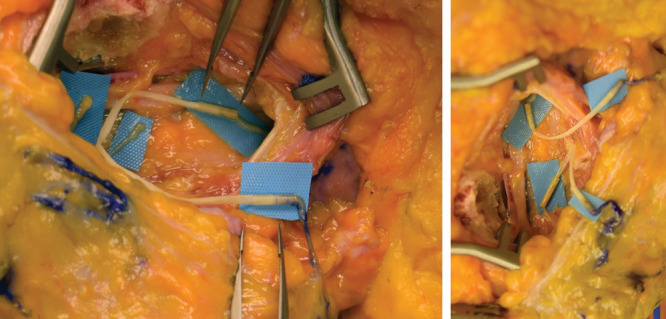
Specimen illustrations of single vs. dual breast neurotization. A, Single breast neurotization between the recipient ICN2 and ICN12, via interposing human allograft. Note spread blue micro pickups pointing to the location of utilized nerve connector facilitating the nerve and allograft coaptation. B, Dual breast neurotization, connecting donor and recipient ICN’s, to optimize neurotization outcomes.

Several cadaveric and clinical studies have shown that the breast is normally innervated via various lateral and cutaneous branches of ICN2 through ICN6.^50–55^ As suggested in the literature, we believe that the ICN3, ICN4, and ICN2 are appropriate recipient nerves for the purpose of flap neurotization. Based on our experience with the cadaver dissections, its identification and dissection are both straightforward and consistent. It is also important to highlight that if dual neurotization is desired, in addition to ICN3, either the ICN2 or ICN4 are also readily available and easily identified within the same surgical field (Figs. 6, 8). Regarding muscular or nerve damage incurred by dissection, the intercostal nerves are already transected during mastectomy and with removal of the breast specimen, so no additional morbidity is incurred by using these nerve stumps. Pectoralis major muscle fibers are spread parallel to their path and do not need to be transected, thus minimizing the muscle damage. This is standard when removing rib cartilage medially and exposing mammary vessels in preparation for microvascular anastomosis.

During the specimen dissection in our study, a 1–2 mm × 50–70 mm interposing allograft was able to easily bridge all nerve gaps between the donor flap and recipient chest nerves. It also allowed free arch of rotation for flap inset and was suitable for either single or dual neurotization. Taking this into consideration and its overall reported effectiveness in nerve reconstruction, allograft appears to offer a solution for current anatomical limitations and thus outcome limitations encountered with the neurotization of DIEP flaps. Future prospective and institutional review board–approved studies have been initiated to help clinically validate allograft effectiveness in breast neurotization. Notably there is no literature available that correlates neurotization rates with postmastectomy radiation, neoadjuvant chemotherapy, and adjuvant chemotherapy. Further research is necessary to assess the effectiveness of neurotization during these circumstances and we would currently recommend against neurotization for these scenarios, given the lack of research and risk of failure. However, a substantial portion of the target population if not the majority will not require postmastectomy radiation.^56^ Barring neoadjuvant or adjuvant chemotherapy, these patients would greatly benefit from neurotization. Another segment of the population that would greatly benefit is the segment undergoing prophylactic mastectomies and thus we believe at baseline there is a significant number of patients that can be helped with neurotization.

There are instances in which spontaneous reinnervation of the flap occurs; however, we believe these cases to be unpredictable exceptions and not the norm. With neurotization, the measure of sensation recovery is more significant and likely occurs under different mechanisms. Although we do not have histological confirmation currently, we believe the reinnervated axons reactivate and supply the original sensory end organs whereas spontaneous regrowth likely produces spontaneous sensation by random ingrowth into the subdermal plexus.

The primary limitation of our dissection findings and the discussed implications is that there have been no clinical studies published, which utilize the selective dissection of only the sensory component of the ICN of the abdominal flap for breast neurotization during DIEP flap reconstructions. However, we are confident that selective dissection and utilization of only the sensory component will significantly improve sensory recovery to be much closer to the sensation of the native breast. Also, we believe that the processed nerve allograft will go hand in hand with this neurotization procedure as the sensory only nerve pedicle will inherently create a nerve gap that is prohibitive of direct neurorrhaphy and bridging with a conduit. The reviewed data are compelling, and we are confident that the utilization of the sensory only component in combination with the allograft for breast neurotization will result in similar outcomes as the referenced nonbreast studies since the principles behind nerve regeneration and the mechanism of regeneration through processed nerve allograft is still the same. In DIEP flap reconstructions, selectively dissecting out and utilizing only the sensory ICN component has the potential to significantly improve sensory recovery and minimize donor-site morbidities.

Another limitation is relatively small study sample (12 hemi-dissections) to suggest appropriate statistical power of observed anatomical variations, thus necessitating prospective clinical evaluations of donor and recipient nerve diameters, their available length upon piercing flap or their distance to flap, all directly affecting the ultimate acquired nerve gap size and thus reconstructive choice. Until those prospective data are available, our data suggest surgeon should be aware of regular nerve variations, potentially affecting what nerve and what reconstructive tool to utilize.

## CONCLUSIONS

Based on our cadaveric dissections, we provide a likely anatomical explanation as to why sensory recovery after current breast neurotization methods during a DIEP flap reconstruction has been less than optimal. The utilization of a mixed sensory and motor nerve autograft is prohibitive to maximal sensory recovery. The sensory branch of the intercostal nerve can be selectively dissected and extracted with the abdominal flap, thus allowing maximal recovery of sensation and preserving the motor branch of the intercostal nerve to the rectus abdominis muscle. Based on these implications, we envision that the integration of this principle will best be served with a processed nerve allograft that has been shown to be effective in neurotization procedures in lengths up to 7 cm. The feasibility and versatility of bridging the existing nerve gap with nerve allograft is demonstrated and the simplicity of the procedure outlined. Ongoing prospective studies are underway to investigate the functional implications of the proposed principle.
